# Australian health policy on access to medical care for refugees and asylum seekers

**DOI:** 10.1186/1743-8462-2-23

**Published:** 2005-10-09

**Authors:** Ignacio Correa-Velez, Sandra M Gifford, Sara J Bice

**Affiliations:** 1 Refugee Health Research Centre, Faculty of Health Sciences, La Trobe University, VICTORIA 3086 Australia; 2 Advocacy Coordinator, Extractive Industries, Oxfam Australia

## Abstract

Since the tightening of Australian policy for protection visa applicants began in the 1990s, access to health care has been increasingly restricted to asylum seekers on a range of different visa types. This paper summarises those legislative changes and discusses their implications for health policy relating to refugees and asylum seekers in Australia. Of particular concern are asylum seekers on Bridging Visas with no work rights and no access to Medicare. The paper examines several key questions: What is the current state of play, in terms of health screening and medical care policies, for asylum seekers and refugees? Relatedly, how has current policy changed from that of the past? How does Australia compare with other countries in relation to health policy for asylum seekers and refugees? These questions are addressed with the aim of providing a clear description of the current situation concerning Australian health policy on access to medical care for asylum seekers and refugees. Issues concerning lack of access to appropriate health care and related services are raised, ethical and practical issues are explored, and current policy gaps are investigated.

## Introduction

Australian health care policy regarding entitlements to medical care for refugees and asylum seekers is complex. On the one hand, health care policy for refugees entering Australia on the offshore humanitarian program is comprehensive, entitling refugees to Medicare, early health assessment, specialised torture and trauma services, and access to the same services as other Australians. On the other hand, health care policy for refugees and asylum seekers who have entered Australia in an unauthorised manner, and who are on a range of visa types, is fragmented. About 40% of asylum seekers living in the community have no rights to access medical care [1].

Health policy for refugee and asylum seekers is directly tied to immigration policy and visa types and this in turn is a complex and rapidly changing field of play. In this paper, we review current health care policies for refugees and asylum seekers in Australia with a focus on those areas of policy gaps that result in a lack of access to medical and health care for some, and less optimal access to care for others. We begin by briefly discussing the definitions of who is deemed to be a refugee and who is deemed to be an asylum seeker, for health care policies vary within and between these two categories accordingly. Second, we describe in some detail, the current health policy for asylum seekers and refugees within Australia's *onshore *program compared to health policy for refugees who have come to Australia through the *offshore *program. Third, we provide a broad comparison of Australian health care policies for refugees and asylum seekers to those of the United Kingdom, Canada and New Zealand, countries with comparable public health systems. Finally, we discuss the current areas where policy on access to health care is in conflict between States and Territories and the Commonwealth and where Australian policy may be in breach of various human rights conventions.

## Who is a refugee or asylum seeker?

Under the United Nations 1951 Convention and 1967 Protocol relating to the Status of Refugees (the Refugee Convention), a refugee is a person who '...owing to a well-founded fear of being persecuted for reasons of race, religion, nationality, membership of a particular social group, or political opinion, is outside the country of his nationality, and is unable to or, owing to such fear, is unwilling to avail himself of the protection of that country' [2]. An asylum seeker is a person who has fled their own country and has sought sanctuary in a second state [3]. They then apply to be recognized as a *bona fide *refugee and to receive legal protection and material assistance that that status implies [4].

Although the 1951 Refugee Convention does not deal specifically with asylum seekers, two of its Articles are particularly relevant to the issues of access to health care considered in this paper. First, Article 33 (*refoulement*) states that no refugee shall be expelled or returned to where his/her life is threatened. Second, Article 31 prohibits punishment or penalties for entry to a state when they come from a territory where life or freedom is threatened. Other aspects of asylum law are particularly relevant to the issue of health policy discussed here. It is important to note that it is not illegal to seek asylum in any country and that the basic provisions related to humane treatment and basic rights apply to asylum seekers. Additionally, International Human Rights Law recognizes the right of all individuals to an adequate standard of living [5].

At the end of 2004 there were an estimated 19.2 million asylum seekers, refugees and other people of concern to the UNHCR [6]. In the same year, 676,000 first instance or appeal applications for asylum were submitted in 143 countries; only 3,276 asylum applications were lodged in Australia, compared to over 500,000 in other industrialized countries [6]. In relation to refugee intake, Australia is one of 16 countries currently participating in the UNHCR-facilitated resettlement program [7]. In 2004, 84,809 refugees were resettled in these countries of which 13,030 were resettled in Australia [8].

## Australian health policy for refugees and asylum seekers: Humanitarian program, health screening and health care access

In order to understand health policies for refugees and asylum seekers, it is important to briefly describe Australia's humanitarian program and the current legislation on visa status and protection. This is because entitlements to health care vary by visa status, and legislation concerning visa status is in a constant state of flux.

### Australia's humanitarian program

Australia's humanitarian program for refugees and others with humanitarian needs includes two components [9]: *offshore resettlement *for people overseas, and *onshore protection *for those who are already in Australia, arrived on temporary visas (e.g. visitor or student visas) or in an unauthorised manner, and are seeking Australia's protection. These two components and their visa categories are shown in Table 1.

**Table 1:** Australia's immigration humanitarian program

	**Visa categories and subcategories**	
	
	**Permanent offshore humanitarian visa categories**	**Temporary offshore humanitarian visa categories**
	
**Offshore resettlement**	• ***Refugee***: for people who are subject to persecution in their home country and who are in need of resettlement •***Special Humanitarian Program (SHP)***: for people who are outside their home country and are subject to substantial discrimination amounting to gross violation of human rights in their home country	• ***Secondary Movement Relocation***: temporary humanitarian visa (THV) to people who have moved from a safe first country of asylum to another country before applying to enter Australia (5-year visa) • ***Secondary Movement Offshore Entry: ***temporary visa to people who arrived without authorisation in Australia at a place outside Australia's migration zone and have moved from a safe first country of asylum (3-year visa)
	**Form of arrival and visas granted**	
	
	**Authorised arrivals**	**Unauthorised arrivals**
	
**Onshore protection**	Most authorised arrivals who subsequently apply for protection receive a bridging visa. The bridging visa allows them to remain in the community while the Protection Visa application is processed. Those who are found to be refugees are granted Permanent Protection Visa (PPV).	Unauthorised arrivals are placed in immigration detention until granted a Protection Visa or removed from Australia. Those who are found to be refugees are granted a 3-year Temporary Protection Visa (TPV).

Sources: [9, 48]

### Policy on health screening for offshore and onshore applicants

Australia's health policy for humanitarian entrants begins pre-arrival. Those who apply under the *offshore resettlement *program must satisfy the health requirement [10] specified in the Migration Regulations. This health requirement, which is set by the Department of Immigration and Multicultural and Indigenous Affairs (DIMIA) on advice from the Commonwealth Department of Health and Ageing, is designed to minimise public health risks to the Australian community, regulate public expenditure on health and community services, and maintain access to health and other services for Australian residents. In general, the health assessment involves a medical examination, a radiological examination to test for Tuberculosis (children under 11 are generally exempt), and HIV/AIDS testing (for all applicants aged 15 or older). For some applicants screening for Hepatitis B is mandatory. Other tests may be requested by a Medical Officer of the Commonwealth.

For those who apply under the *onshore protection *program, the health screening varies depending on their specific circumstances. In general, those who arrive in Australia on a 3-month temporary visa – or less – are not required to have formal health examinations prior to their arrival (some exceptions apply [11]). Those arriving on a greater than 3 months temporary visa may be required to undergo formal medical and radiological examinations prior to arrival, depending on the level of health risk of their country of origin, their age, and the purpose of their stay (e.g. likely to enter a hospital, health care area, classroom, preschool or childcare centres).

All unauthorised arrivals who are applying for Australia's protection undergo health screening soon after their arrival at immigration detention centres. "Where any serious communicable diseases are suspected or confirmed, formal notification procedures are followed with Commonwealth and State/Territory health authorities" [12] (p. 2). In general, those who make an onshore application for protection, whether arriving on an authorised or unauthorised manner, are required to undergo medical and radiological examinations before the granting of the visa.

According to DIMIA, the only health condition that formally precludes the grant of a visa is active or untreated tuberculosis (TB) [10]. Those applicants whose TB has been treated or those with a previous but currently non-active TB, are required to make a Health Undertaking. In other words, they are required to contact the Health Undertaking Service on arrival in Australia, and to report to State or Territory health authorities for follow-up assessment [10]. The Health Undertaking also applies for a pregnant applicant who has not had the chest X-ray as part of the standard health examination (although this is not commonly extended to persons from high risk TB countries).

All other health conditions are assessed on a case by case basis taking into account the risk to public health, the estimated costs, and the resource use impact on the Australian community. The final authority as to whether the health requirement is met rests with the Medical Officer of the Commonwealth (MOC). The visa processing officers are required to accept the opinion of the MOC. However, where there are compelling factors, the Minister's delegate processing officer may waive the health requirement for refugees and other humanitarian visa applicants. If the applicant or any member of their family fails to pass the health requirement, the entire family group can be denied a visa. They may however, be referred to other resettlement countries such as the United States, who have different health screening guidelines and selection criteria. For those applying under the *onshore protection *program, they may be denied a protection visa should they fail the health requirement under the same above considerations.

### Policy on medical care for offshore and onshore applicants

While Australian policies on resettlement of humanitarian refugees are arguably among the best, compared to other United Nations High Commissioner for Refugees (UNHCR) resettlement countries, its policies on the treatment of onshore protection applicants have been strongly criticised by human rights organisations, scholars, government members and others for their denial of basic human rights guaranteed under the 1951 Refugee Convention [2]. A brief description of the medical care entitlements granted to different categories of offshore humanitarian entrants and onshore protection applicants is given in Table 2.

**Table 2:** Health entitlements commonly granted to refugees and asylum seekers in Australia

**Humanitarian program**	**Circumstances**	**Entitlements**
**Offshore resettlement**	Refugees who hold permanent offshore humanitarian visas	Same eligibility for Medicare and Health Care Card, including Pharmaceutical Benefits Scheme, as other permanent residents. Eligible for Early Health Assessment and Intervention Program and torture/trauma services.
	Refugees who hold temporary offshore humanitarian visas (THV)	Able to gain access to Medicare and Health Care Cards, including Pharmaceutical Benefits Scheme; eligible for referral to the Early Health Assessment and Intervention Program and torture/trauma services.
**Onshoore protection**	**Authorised arrivals**	
	Authorised arrivals who have been found to be refugees and are granted permanent protection visa (PPV)	Same eligibility for Medicare and Health Care Card, including Pharmaceutical Benefits Scheme, as other permanent residents.
	Authorised arrivals who applied for protection within 45 days of their arrival in Australia and are awaiting primary decision on their application	Eligible for Medicare ^a^
	Authorised arrivals who have been in Australia for 45 days or more before they applied for protection	No access to Medicare ^a^
	Authorised arrivals who have appealed or are about to appeal to the Refugee Review Tribunal or the Administrative Appeals Tribunal after their primary protection application has been refused	No access to Medicare ^a^
	Authorised arrivals who are appealing to the Minister of Immigration after being found not to be refugees at the review stage	No access to Medicare ^a^
	**Unauthorised arrivals**	
	Unauthorised arrivals who have been found to be refugees and are granted temporary protection visa (TPV)	Access to Medicare benefits and Health Care Card; eligibility for torture and trauma counselling
	Unauthorised arrivals who are applying for protection and are in immigration detention	Access to health care through health professionals contracted by the private company in charge of the detention centres
	Unauthorised arrivals who have been in mandatory detention, are subsequently released into the community, and have an outstanding visa application	No access to Medicare
	**Others**	
	Asylum seekers who hold or held a TPV or a THV, have had their application for further protection finally refused, have exhausted all legal options to remain in Australia and are making arrangements for departure (return pending visa)	Eligible for Medicare access through work rights, Health Care Card, including Pharmaceutical Benefits Scheme, torture/trauma counselling, Maternity Allowance, maternity immunisation
	Asylum seekers who have been held in detention, do not have any outstanding visa applications or litigation, who cannot currently reasonably be removed from Australia, and who agree in writing to cooperate with their removal from Australia when advised that they must leave (removal pending bridging visa) ^b^	Eligible for Medicare access through work rights, Health Care Card, including Pharmaceutical Benefits Scheme, torture/trauma counselling, Maternity Allowance, maternity immunisation

Sources: [15, 49-54]

^a ^Under certain circumstances, these individuals may be eligible for the Asylum Seeker Assistance Scheme (ASAS) [21]

^b ^It can apply to both unauthorised arrivals held in detention or authorised arrivals who are in detention after breaching their visa conditions

#### Offshore resettlement program

On arrival to Australia, all refugees and special humanitarian program (SHP) entrants under the *offshore resettlement *program receive all the entitlements granted to Australian permanent residents, including access to social security benefits (Centrelink), Medicare, education and training (including 510 hours of English language lessons), and employment services. In addition, they receive settlement support through the Integrated Humanitarian Settlement Strategy (IHSS), which is carried out by agencies responsible for a range of settlement services. The IHSS includes [13]: initial information and orientation assistance on the essential services available, accommodation support, household formation support, early health assessment and intervention, and community support. The early health assessment takes places within the first 12 months of arrival, and involves information on available health services, physical health and psychological/psychosocial assessments, and referral to other health services where required (including torture/trauma services). A current policy gap however, relates to early health screening for infectious diseases.

In the 1970's and 80's, all new arrivals under the *offshore *program were offered health screening and assessments which included immunization updates, screening for parasitic and infectious diseases. This was more easily accomplished as new arrivals spent the first few weeks within a migrant hostel. However, in the 1990's Commonwealth policy shifted so that new arrivals were settled directly into the community along with the expectation that early health assessments would be carried out by local general practitioners. Thus, there is at present no comprehensive policy on health screening for new arrivals. Further, there is considerable debate about the merits of bringing in compulsory health screening or whether this is better carried out within a more holistic approach allowing people to access screening throughout the resettlement period [14].

#### Onshore protection program

Our discussion on Australia's policy on medical care within the *onshore protection *program begins with those asylum seekers in detention centres. The immigration detention policy, introduced in 1992, has been maintained with bipartisan support in Parliament [15]. The Commonwealth, not the States and Territories, is responsible for the health care of detainees. The private company, Global Solutions Limited (Australia) Pty Ltd, contracted by the Commonwealth to manage the detention centres, is responsible for providing healthcare to all detainees. It employs nurses, general practitioners and psychologists [16]. According to the Commonwealth Government, detained asylum seekers have '24 hour medical services, dental services, culturally responsive physical and psychological health services' [15]. Inquiries carried out by the Human Rights and Equal Opportunity Commission (HREOC) into the detention centres, however, report serious concerns in terms of the adequacy and quality of health care services available for the detainees, particularly the failure to diagnose and treat torture/trauma survivors [17,18].

Although attention is often focussed on Australia's detention policies, Australian policy can also be criticised for infringing the rights of asylum seekers through denial of access to appropriate health care and related services [19]. The three case studies below illustrate the complexities of current policies relating to the provision of health care for asylum seekers and Temporary Protection Visa (TPV) holders in Australia (NB: names have been changed).

##### Case 1

*Ms Jayawardene and her husband have been living in Australia as asylum seekers for the past year. They are currently on Bridging Visa E, which allows them to live in the community but gives them no work rights, no entitlement to Medicare and no access to any social security benefits. Ms Jayawardene has recently given birth. She had no prenatal care. Only after a caseworker from a community based organisation (CBO) advocated on her behalf, was she hospitalised for the birth. She was, however, discharged early because she was unable to pay the fees. The hospital only agreed to waive the fees after much negotiation, carried out on behalf of the couple by the CBO*.

##### Case 2

*Mr Hassan sought asylum in Australia two years ago, after having spent several years living in a number of different countries. Mr Hassan spent one year in mandatory detention before he was deemed a refugee and granted a three-year Temporary Protection Visa (TPV), which confers on him a range of entitlements, including Medicare. He recently consulted a local general practitioner to seek treatment for a stomach ailment, but was unable to properly communicate about his symptoms due to his lack of English language skills. Although Mr Hassan is entitled to Medicare benefits, he is not entitled to other settlement services, such as adequate English-language tuition or fee-free interpreting services. The lack of access to an interpreter created a barrier to Mr Hassan receiving a proper health assessment*.

##### Case 3

*Mr Ahmed has a serious chronic heart condition that requires medication. He waited six months for a primary decision on his application for protection before he qualified for the Asylum Seeker Assistance Scheme (ASAS)-funded by DIMIA- which assists with basic needs, including health care costs. Two months ago, however, Mr Ahmed lost access to ASAS because he appealed a negative Refugee Review Tribunal (RRT) decision on his application for refugee status. He is now unable to pay for essential medication and relies on charitable assistance to provide his medication*.

These three case studies illustrate a number of key areas where access to health care is either denied or inadequately provided to people residing in Australia, and raise a number of questions about health policies for *onshore protection *applicants and TPV holders.

##### Community based onshore protection applicants

Access to health care for asylum seekers living in the community depends on two elements: the type of bridging visa they hold and the particular stage of their application [20].

Until 1997, most *onshore protection *applicants were granted a bridging visa after their original visa (e.g. a visitor visa or student visa) had expired. With some exemptions, this bridging visa conferred work rights, which consequently entitled most holders of the visa to Medicare, the Australian Government health insurance scheme. On 1 July 1997, however, the government introduced work rights regulations for asylum seekers who applied for a protection visa (PV) on or after that date. According to these regulations, 'a bridging visa with work rights may be granted to people who have been in Australia for fewer than 45 days in the 12 months before they lodge a PV application [21]'. In other words, those asylum seekers who have been in Australia for 45 days or more in the 12 months before they make the PV application are not permitted to work, and therefore, do not have access to Medicare. It has been reported that this requirement has resulted in approximately 40 per cent of community based asylum seekers being denied Medicare [1]. Usually, these asylum seekers are granted a Bridging Visa E.

From 1 January 2001, PV applicants who 'have ever applied (on- or off-shore) for a parent visa, irrespective of whether their application is on-hand, finally determined or withdrawn, have no access to Medicare [21]'. For individual asylum seekers with no work rights, the 'no work' condition may only be changed if DIMIA has not made a primary decision within six months of the lodgement of their application and the applicant demonstrates a compelling need to work [21]. In some circumstances, work rights and Medicare may also be available to asylum seekers if they are the spouse, child or parent of an Australian citizen or permanent resident.

In an attempt to fill this welfare gap, DIMIA administers the Asylum Seeker Assistance Scheme (ASAS) through contractual arrangements with the Australian Red Cross. Operating since 1993, ASAS was designed to financially assist asylum seekers who were unable to meet their most basic needs (e.g. food, accommodation, health care). ASAS recipients can receive assistance with health care costs and referral to counselling services.

Initially, all asylum seekers were entitled to ASAS at the primary and review stages of their application, if a decision had not been made within six months. The eligibility criteria for ASAS, however, have been gradually restricted. Currently, to be eligible for ASAS, asylum seekers must be in financial hardship and: have lodged a valid PV application for which a primary decision has not been made within six months; not be in detention and must hold a bridging or other visa; not have been released from detention on an undertaking of support; not be eligible for either Commonwealth or overseas government income support; and not be a spouse, *de facto *or sponsored fiancé(e) of a permanent resident [21]. There are some exemptions to the above criteria, such as: unaccompanied minors or elderly persons (over 65 years); parents with children under 18 years of age; women with high risk pregnancies; and persons who are unable to work as a result of a disability, illness or torture/trauma [21].

ASAS may also be extended to RRT applicants who are unable to meet their basic needs and who have no adequate support [21]. There have been a few cases in which DIMIA has used its discretionary powers to continue providing certain asylum seekers, who are experiencing exceptional welfare circumstances, with 'special payments' while their cases are at the post-RRT stage [19]. However, once the RRT makes a decision on the application, most asylum seekers cease to be eligible for ASAS [21].

In March 2005, the Minister for Immigration and Multicultural and Indigenous Affairs announced the creation of the Removal Pending Bridging Visa (RPBV), intended to 'provide greater ability to manage the cases of long term detainees who are awaiting removal' [22]. The visa allows a relatively small number of asylum seekers – those who have exhausted all legal appeals, but who cannot be reasonably removed from Australia, and who are willing to agree in writing that they will leave Australia when instructed to do so by the government – to enter the community. These asylum seekers are eligible for Medicare, through work rights, and have access to Health Care Cards, including the Pharmaceutical Benefits Scheme, maternity care and torture/trauma counselling.

More recently (21 June 2005) the Federal Government introduced further changes to detention which will include, among others, the release from detention of children and their families into the community [23]. Under these arrangements, DIMIA is funding the Red Cross to develop a national system that will provide material and health care support for those asylum seekers released into the community [24].

These recent changes, including the introduction of the RPBV, add another significant layer of complexity to an already complicated system. Questions raised by the RPBV visa and its potential implications for asylum seekers are discussed later in the paper.

##### Temporary Protection Visa holders

According to current migration regulations, refugees on TPV have access to Medicare, are eligible for referral to the Early Health Assessment and Intervention Program (EHAI), and for torture/trauma counselling [25]. TPV holders, however, face serious challenges when accessing health care services. For instance, those over the age of 18 are not eligible for government-funded English language classes. Low levels of English language skills cause social isolation, unemployment (or low paid employment and therefore inability to afford medications), higher incidence of occupational health and safety issues, and obvious difficulties when accessing health care services. TPV holders are not allowed to access the federally funded Telephone Interpreting Service or the various health interpreting services designed to assist health workers who treat people from non-English Speaking backgrounds. In addition, amputees on TPV are not able to access the Commonwealth Rehabilitation Service [26].

In early July 2004, the government introduced new measures for TPV and Temporary Humanitarian Visa (THV) holders [27]. Briefly, these changes include a reintegration assistance package for TPV and THV holders who are prepared to return to their countries, the introduction of a new 'Return Pending Visa', which will allow those TPV holders whose applications for further protection has been rejected to stay in Australia for 18 months while they prepare for departure; and changes to enable TPV and THV holders to apply for a range of non-humanitarian onshore visas to live permanently in Australia. These recent developments, however, do not represent major changes in health care access and other entitlements to TPV holders.

Despite the presence or absence of health policies relating to entitlements to medical care, there are a range of barriers to accessing health care services, common to most refugees, independent of their visa status. Some of the barriers that have been identified are:

• Long waiting times particularly in using Emergency Departments of public hospitals,

• Cost of services, especially for specialist health care and in relation to public dental health services,

• Lack of information and confusion about the health system, particularly the difference between public and private and entitlements,

• Lack of interpreters and female physicians, particularly in rural areas,

• Absence of bulk billing services in rural areas,

• Instances of discrimination,

• Other settlement needs taking precedence, particularly in cases where refugees are employed in casual or temporary work with no leave provisions,

• Lack of specialist care, particularly in regional areas [28].

These barriers are of particular concern as most refugees arrive in Australia in poor health and are likely to face particular health challenges in the resettlement period. These health challenges stem from previous experiences of torture and trauma and from having lived in poor social and economic prior to arrival, all of which impact on their well-being during the resettlement period [14].

## A comparison of health policy for refugees and asylum seekers across selected industrialised countries

Australia is one of sixteen countries who accept refugees for resettlement under the UNHCR resettlement program [29]. Full entitlement to Medicare and additional entitlements to health care for special needs, including oral health and mental health, are also provided by the Commonwealth, State and Territory governments [21]. In fact, Australian health care policy for newly arrived humanitarian entrants is comprehensive and one of the best compared to other resettlement countries [30]. When it comes to onshore protection applicants, however, at the time of this writing Australia compares poorly to other resettlement countries.

While entitlements to social benefits and health care vary in the other resettlement countries, at the time of this writing, none of these countries deny asylum seekers the right to basic medical care. Indeed, a brief review of the refugee and asylum seeker health policies of the United Kingdom (UK), Canada and New Zealand indicates that Australian policy is comparatively lacking in this respect (See Table 3).

**Table 3:** Country comparison of health entitlements for refugees and asylum seekers according to status

	**Australia**	**New Zealand**	**Canada**	**UK**
**Refugee**	Medicare	NZ Health System coverage	Provincial Health Cover: Canada's Federal Health system (comprised of Federal and Provincial contributions) includes comprehensive health cover, including hospital, physician, surgical-dental and specialist cover.	NHS Coverage
**Asylum Seeker in detention**	Health care through the private company in charge of the detention centres ^a^	N/A: Asylum Seekers rarely held in detention for longer than 48 hours ^b^	No overarching coverage: individuals are assessed on a case-by-case basis ^b^	National Health Service (NHS) Coverage. Coverage includes: primary & secondary care, free prescriptions, dental services, coverage of travel costs to/from hospital ^b^
**Asylum Seeker awaiting primary decision of refugee status**	Depends on when visa applied for: If application submitted within 45 days of arrival, then individuals have access to Medicare but no translating services, Early Health Assessment and Intervention, and torture/trauma counselling, If application submitted after 45 days of arrival, then no Medicare access	NZ Health System coverage. Coverage includes all services, such as: Primary & secondary care, co-payment of pharmaceuticals, specialist referral and coverage, cost offsets for 'frequent users' of medical services, hospital & accident cover, dental, mental, maternity and sexual health care.	Interim Federal Health (IFH) Program coverage. Coverage includes essential health services for the treatment & prevention of serious medical conditions, essential prescription medications, contraception, prenatal care, obstetrical care, Immigration Medical Exam, emergency dental service.	NHS coverage
**Asylum Seeker appealing a negative Refugee Review Tribunal (or equivalent) outcome**	No access to Medicare ^c^	NZ Health System coverage NB: may take some time to receive Community Services Card, necessary for accessing a General Practitioner (GP)	IFH Program Coverage	NHS Coverage
**Refused Asylum Seeker who has exhausted all appeals**	No access to Medicare ^d^	NZ Health System coverage NB: may take some time to receive Community Services Card, necessary for accessing a GP	IFH Program Coverage	Primary & urgent care only ^e^

Sources: [14, 31-33, 36, 37, 49, 55-60]

^a ^Detention is mandatory for all 'unauthorised arrivals'.

^b ^Detention is *not *mandatory for 'unauthorised arrivals'.

^c ^Under certain circumstances, these individuals may be eligible for ASAS. See: DIMIA, 2003.

^d ^The majority of individuals in this circumstance will not have Medicare access. A small number of individuals living in the community on 'Removal Pending Bridging Visas' will, however, have access.

See: Table 2 for further explanation.

^e ^Asylum Seekers who are deemed 'hard cases' maintain NHS coverage until a decision has been reached.

In Canada, for example, asylum seekers – including those facing deportation – are entitled to primary and emergency care, essential health services for the treatment and prevention of serious medical conditions, essential prescription medications, and prenatal and obstetrical care, among others. Refugees and asylum seekers may also receive secondary and mental health care with prior approval [31,32].

The UK offers similar coverage for refugees and asylum seekers, providing access to a broad range of National Health Service (NHS) benefits, including primary and secondary care, optical and dental care, free prescriptions and coverage of travel costs to/from hospital [33]. It is important to note that recent changes to UK law suggest a tightening in the NHS' willingness to provide such broad services across all asylum seeker categories [34]. Failed asylum seekers who have exhausted all rights of appeal are now only eligible for urgent care at no cost [35]. Despite this reduction in the range of available entitlements, the UK's policy still offers greater medical coverage than that available to many asylum seekers living in the community in Australia.

New Zealand, Australia's closest resettlement neighbour, provides refugees with the same health services as residents through the Publicly Funded Health and Disabilities (PFHD) Service. Asylum seekers with applications pending also have access to these services through a Community Services Card [36]. Other asylum seekers, for example those who are appealing a negative Refugee Status Appeal Authority decision, also have access to PFHD services [37].

## Grey areas: complexities of the Australian health care policy for refugees and asylum seekers

The sheer range of diverse and complicated refugee and asylum seeker visa types exemplifies the complexity of Australian health care and health policy for refugees and asylum seekers. This complexity often leads to confusion among refugees, asylum seekers, community workers and health care practitioners alike. Additionally, current health care policy presents numerous grey areas. In particular, problems exist around gaps between the legal and practical applications of these policies; lack of policy coordination between State and Commonwealth governments; asylum seekers' ability to successfully access necessary health care; decisions about granting ASAS; lack of publicly accessible government data; and implications of the new RPBV.

Significant gaps between legal and practical policy implementation and the lack of coordination between State and Commonwealth governments may be best exemplified by the issue of access to public hospitals for asylum seekers. To begin, it is important to distinguish between legal restrictions and *de facto *restrictions. Disparities between being eligible to access health care and being able to access health care epitomise these gaps. In other words, asylum seekers with no Medicare can, theoretically, access public hospitals but may not be able to do so because they fear that their lack of income will leave them unable to pay hospital fees. There is growing evidence suggesting that Medicare ineligible asylum seekers have been turned away from hospitals, have not completed their required medical treatment, or have been asked to pay outstanding hospital bills and this is of significant concern [20].

Although some State governments have attempted to improve asylum seekers' access to health care and welfare [38], the Catholic Commission for Justice Development and Peace reports that State governments have failed 'to provide clear instructions to their departments and agencies to protect the human rights of asylum seekers in the areas of housing, health, transport and education' [39]. Additionally, State and Territory governments do not have clear policies concerning Medicare ineligible asylum seekers. In New South Wales (NSW), for example, an assurance of payment is required before treatment will be provided. If, however, that assurance is not available, then patients will 'receive only the minimum and necessary medical care to stabilise their condition' [1].

Strategies and practices for the provision of care to this population also vary widely across public health care services. While a few services provide ease of access to asylum seekers, many CBOs report that the majority deny access or attempt full fee recovery after providing the services. Commonly, access to these services, including waiving of fees, is dependent on long term advocacy from CBOs [19].

Other factors, such as English language skills and ability to access transport, also influence asylum seekers' ability to access successfully health care, thereby creating even greater gaps between policy and practice. As mentioned previously, current Commonwealth policy does not provide asylum seekers on Bridging Visas and refugees on TPV access to English language tuition or fee-free interpreting. Studies from the UK indicate that misunderstandings and poor communication between medical practitioners and asylum seekers operate as barriers to appropriate health care [1]. Clearly, this is also the case for refugees and asylum seekers within Australia, who do not have access to fee-free interpreting services.

Transport is often another major barrier to refugees and asylum seekers attempting to access health care with many lacking the money necessary for public transport or taxis. Could they access a vehicle, many are ineligible for drivers' licences, unless they can read, write and understand English sufficiently to pass the exam.

A key barrier towards improving health policy for refugees and asylum seekers is the difficulty in obtaining clear information from relevant Commonwealth Departments. For example, in relation to ASAS eligibility, it is unclear how DIMIA applies its discretionary powers to extend eligibility to ASAS or similar 'special payments' to some asylum seekers whose cases are at the post-RRT stage. Similarly there is a lack of publicly available government data on several key issues regarding asylum seeker health policy. First, it is unclear how many asylum seekers are living in the community on Bridging Visa E. This makes it difficult for CBOs to assess levels of need and asylum seeker populations. Second, there is currently no procedure for recording asylum seekers' access to health care which has lead to a lack of knowledge to inform policy and practice. Third, there are no available data for analysing the numbers of refugees and asylum seekers using public hospital services and, finally, there are no data on what they are being treated for. These issues, in turn, make it virtually impossible to estimate rates of and reasons for admission. Thus, we are left with case studies and documentation carried out by the already strapped CBOs working in the sector.

The recent introduction of RPBV only complicates these grey areas further. In particular, the RPBV creates disparities between bridging visas, presents significant human rights issues and has the potential to lead to further mental health issues for these asylum seekers. First, the RPBV's provision of Medicare raises significant issues around the levels of health care access for asylum seekers on other types of Bridging Visas. For example, what is the rationale behind the decision to grant Medicare rights to this Bridging Visa, when existing Bridging Visas offer no similar rights? Why has the provision of greater rights under this Bridging Visa not translated to other, similar visa categories? That is, if Medicare has been deemed necessary for these Bridging Visa holders, why has this not been extended to other Bridging Visa holders?

The RPBV also raises great concern about potential denial of human rights. Currently, the RPBV requires asylum seekers to agree in writing that they will cooperate with their removal from Australia when the government deems it is safe to do so. Thus, there is the very real potential for the promise of release from detention to lead to asylum seekers relinquishing their legal rights and future opportunities for visas. The written agreement may also facilitate involuntary return – individuals will have no say about the safety of their return, once the government deems it should happen [40].

The RPBV may also have significant effects on the mental health of asylum seekers. There is a growing body of evidence on the negative mental health impacts of detention on asylum seekers [41,42]. Releasing these already traumatised individuals into the community without immediate and ongoing access to counselling, and with no definite return date, no guaranteed visa term and no rights of appeal, could lead to further mental health issues, such as depression, feelings of isolation and anxiety.

## Filling the Gaps: the need to adopt minimum standards

Current Australian health policy on access to medical care for refugees and asylum seekers has two faces. On the one hand, health care access for resettling refugees is comprehensive and one of the best compared to other resettlement countries. On the other hand, health policy for asylum seekers is less than adequate to ensure a minimum standard of health care. Moreover, the gaps between Commonwealth policy and State and Territory policy has produced a climate of confusion especially in regards to who pays.

As Taylor argues, current Australian policy for asylum seekers is 'insufficient to assure them of an adequate standard of living', and in breach of the International Covenant on Economic, Social and Cultural Rights (ICESCR) [43]. The current policy context may also leave Australia in breach of other 'international legal conventions and recommendations regarding its obligations toward asylum seekers living in its territory' [19] (p.16). Expanding on the human rights perspective, Dwyer argues that refugee and asylum seeker health care is also an issue of social justice and social responsibility [44]. In order to meet its basic human rights, social justice and social responsibility requirements, therefore, Australian health policy for asylum seekers needs significant and immediate change.

There are two key areas where policy reforms are urgently required. First, Commonwealth and State and Territory health care policy for asylum seekers needs to be coordinated to remove the current gaps which result in little or no access to health care for a proportion of the asylum seeker population [14]. Second, serious consideration should be given to extending medical care to all asylum seekers residing on Australia soil, regardless of their visa status. This medical care should include at least primary medical and psychological health care and care that cannot be postponed [45]. While there has been no rigorous economic analysis of the potential costs of extending Medicare to the entirety of this population, a small study indicates that the costs would be about A$ 2.9 million per year for NSW [46]. A similar analysis conducted by one of the authors (I C-V) estimated that the expected health care costs for asylum seekers living in the Victorian community would be about A$1.5 million per year. This figure represents about 0.009% of the total annual health care expenditure in Victoria in 2000–2001 [47]. These two initiatives would go a long way towards health policy reform for this vulnerable, high need but numerically small population residing in Australia.

## List of abbreviations

ASAS: Asylum Seeker Assistance Scheme

CBO: Community based organisation

DIMIA: Department of Immigration and Multicultural and Indigenous Affairs

EHAI: Early Health Assessment and Intervention Program

HIV: Human Immunodeficiency Virus

HREOC: Human Rights and Equal Opportunity Commission

ICESCR: International Covenant on Economic, Social and Cultural Rights

IFH: Interim Federal Health

IHSS: Integrated Humanitarian Settlement Strategy

MOC: Medical Officer of the Commonwealth

NHS: National Health Service

PFHD: Publicly Funded Health and Disabilities

PPV: Permanent Protection Visa

PV: Protection visa

RPBV: Removal Pending Bridging Visa

RRT: Refugee Review Tribunal

SHP: Special Humanitarian Program

TB: Tuberculosis

THV: Temporary Humanitarian Visa

TPV: Temporary Protection Visa

UNHCR: United Nations High Commissioner for Refugees

## Competing interests

The author(s) declare that they have no competing interests.

## Authors' contributions

All authors contributed equally to this paper.
